# Inflammation is associated with altered functional connectivity of the cingulate cortex in individuals with subthreshold depression

**DOI:** 10.1371/journal.pdig.0001665

**Published:** 2026-09-17

**Authors:** Zhangzhang Qi, Pan Chen, Guanmao Chen, Zhenye Luo, Shu Zhang, Lijun Jiang, Guixian Tang, Li Huang, Qian Tao, Ying Wang

**Affiliations:** 1 Medical Imaging Center, First Affiliated Hospital of Jinan University, Guangzhou, China; 2 Institute of Molecular and Functional Imaging, Jinan University, Guangzhou, China; 3 Department of Public Health and Preventive Medicine, School of Basic Medicine, Jinan University, Guangzhou, China; 4 Division of Medical Psychology and Behavior Science, School of Basic Medicine, Jinan University, Guangzhou, China; CYENS Centre of Excellence, CYPRUS

## Abstract

Subthreshold depression (StD) represents a critical prodromal phase that often precedes clinical depression. Identifying biological indicators during this stage is essential for the prophylaxis of major depressive disorder. The present study investigated the interplay between serum pro-inflammatory cytokines, functional connectivity (FC) within subregions of the cingulate cortex (CC), and psychometric profiles in an StD cohort. The study included 126 participants with StD and 104 healthy controls. Resting-state functional magnetic resonance imaging data, psychological assessments [Center for Epidemiologic Studies Depression Scale (CES-D), 24-item Hamilton Depression Rating Scale (HAMD-24), Hamilton Anxiety Scale (HAMA), Ruminative Responses Scale (RRS)], and serum pro-inflammatory cytokine levels [tumor necrosis factor-alpha (TNF-α), interleukin-1 beta (IL-1β), IL-6, and IL-17] were collected. The StD group exhibited increased FC in three specific circuits: The left middle CC (MCC) to the bilateral caudate nucleus (CN); the left supracallosal anterior CC (supACC) to the bilateral anterior cerebellar lobe; the right supACC to the right superior frontal gyrus (SFG). Furthermore, serum concentrations of TNF-α, IL-1β, and IL-17 were significantly increased. FC between the left MCC and bilateral CN was associated with CES-D and RRS scores, whereas FC between the right supACC and right SFG was associated with HAMA scores. Interaction between IL-1β and FC in the right supACC-SFG circuit was independently associated with HAMA scores, whereas interaction between IL-17 and FC in the same circuit independently predicted RRS scores. The statistical interaction between IL-1β and IL-17 levels and right supACC-SFG FC is associated with anxiety and rumination symptoms in StD, which may indicate an increased risk of progression to major depression. These findings underscore the significance of neuroimmune associations in subthreshold depression.

## Author summary

Subthreshold depression (StD) is a common early stage of depression, marked by low mood and anxiety that fall short of a full diagnosis. People with StD are at higher risk of developing major depression, yet we lack simple biological signs to identify this early stage and guide prevention. In this study, we combined brain imaging with blood-based markers to investigate how brain function and inflammation relate to StD symptoms. We studied 126 young adults with StD and 104 healthy volunteers. Using resting-state brain scans, we measured communication between brain regions in the cingulate cortex, an area important for mood and thinking. We also measured four immune signaling molecules (pro-inflammatory cytokines) in the blood. Compared with healthy volunteers, the StD group showed stronger connections between the cingulate cortex and three brain regions involved in emotion and self-control, together with higher blood levels of three cytokines. Notably, the combined effect of two key cytokines (IL-1beta and IL-17) and altered brain connectivity predicted anxiety and repetitive negative thinking (rumination) in StD. These results link immune changes to early brain alterations in depression and point to a neuroimmune signature that could help identify people at risk and support early, digital-health-based prevention.

## 1 Introduction

Often termed subclinical, minor, or subsyndromal depression, Subthreshold depression (StD) is characterized by clinically significant depressive symptoms that do not meet the full diagnostic criteria for major depressive disorder (MDD) [1]. This operational definition is consistent with common conceptualizations of subthreshold conditions in the literature, including subsyndromal depression, minor depression, and subthreshold depressive symptoms [1,2]. Functioning as an intermediate state, StD exhibits a bidirectional trajectory: it may progress into a major depressive episode or improve following targeted intervention [3,4]. Consequently, StD is considered an indicator of depression risk. Depressive and anxiety symptoms are strongly associated with self-reported rumination, which is a robust predictor of future depression [5]. Compared with MDD, StD has a higher prevalence; however, it is frequently overlooked because the severity is relatively mild. Individuals at greatest risk of progressing from StD to major depression would derive substantial benefit from timely intervention [6]. Therefore, identifying biological markers of StD is essential for depression prevention.

Functional [7], metabolic [8], and volumetric [9] abnormalities of the cingulate cortex (CC) have been frequently documented in depression research. CC is interconnected to multiple brain regions and plays a critical role in cognitive control processes [10,11]. Anatomically, CC is divided into three major subdivisions: the anterior CC (ACC), middle CC (MCC), and posterior CC (PCC) [12]. Located at the convergence of the corticolimbic network, ACC maintains extensive bidirectional connections with the prefrontal cortex and is essential for integrating emotional and cognitive information [13]. The functional role of MCC remains under debate; however, previous evidence has associated it with activation during goal-directed behaviors [14]. PCC, a central component of the default mode network, demonstrates widespread connectivity throughout the brain and contributes substantially to cognitive control, with functional variations associated with learning, memory, and task engagement [15]. Although meta-analyses of regional cerebral function indicate that CC dysfunction plays a substantial role in the pathophysiology of depression [16], studies specifically investigating CC function in StD remain limited. Our previous functional MRI study identified differential activations across various brain regions, including CC, when comparing individuals with StD to healthy controls (HCs). These findings highlight the neural mechanisms underlying negative emotion processing in StD [17]. Li et al. have reported alterations in local activity and functional connectivity (FC) within the ACC among older adults with StD [18]. Nonetheless, the specific contribution of FC abnormalities across CC subregions in StD remains insufficiently understood.

Evidence indicates that peripheral inflammation contributes significantly to depression pathophysiology [18]. Pro-inflammatory cytokines, including tumor necrosis factor-α (TNF-α), interleukin-1β (IL-1β), interleukin-6 (IL-6), and interleukin-17 (IL-17), can cross the blood-brain barrier and influence brain function. These cytokines have been associated with sickness behaviors and depressive symptoms in physically ill individuals without a prior history of psychiatric disorders [19–22]. Furman [23] linked cytokine-induced mood alterations to connectivity between the nucleus accumbens and ACC. The present study investigated neural mechanisms underlying inflammation-associated alterations in CC FC in StD.

This study employed rs-fMRI to assess whole-brain seed-based FC across CC subregions in homogeneous cohorts of individuals with StD and HCs. Blood samples were collected to quantify TNF-α, IL-1β, IL-6, and IL-17 concentrations and to explore the relationship between inflammatory cytokine levels and FC alteration in CC subregions among individuals with StD. We hypothesized that individuals with StD would exhibit abnormal FC patterns across CC subregions and an altered pro-inflammatory cytokine profile, with CC dysfunction associated with cytokine alterations.

## 2 Materials and methods

### 2.1 Ethics Statement

The Ethics Committee of the First Affiliated Hospital of Jinan University, China, approved the study (Approval No. 028, 2019). All participants provided written and verbal informed consent after receiving a comprehensive explanation of the study.

### 2.2 Participants

We recruited students diagnosed with StD from Jinan University and other universities in Guangzhou City via digital and print advertising from March 2019 through June 2023. Subthreshold depression (StD) was operationally defined in accordance with previous widely accepted criteria [1,2]: ① the presence of at least one core depressive symptom (depressed mood or anhedonia);② clinically significant depressive symptoms that do not meet DSM‑5 diagnostic criteria for major depressive disorder; and③mild‑to‑moderate psychosocial functional impairment. Eligibility requirements included: right-handedness, Han ethnicity, and an age range of 18–35 years, with a Centre for Epidemiologic Studies Depression Scale (CES-D) [24] score of 16 or above, a 24-item Hamilton Depression Rating Scale (HAMD-24) [25] score between 8 and 20 indicating mild depression, and experiencing nonseasonal depressive symptoms. Exclusion criteria included the Diagnostic and Statistical Manual of Mental Disorders (DSM–5) diagnoses of bipolar disorder, major depressive disorder, schizophrenia, or other mental disorders; antidepressant use in the past year; suicidal thoughts or actions; MRI contraindications; pregnancy or breastfeeding; recent acute infection, allergy, autoimmune diseases, obesity, current smoking, or alcohol abuse; and other serious diseases needing treatment. Participants in the HC group were identified by scoring below 16 on the CES-D and below 8 on the HAMD-24 during both assessments. Participants underwent the Structured Clinical Interview for DSM-5 Nonpatient Edition (SCID-NP) to exclude any personal or first-degree familial psychiatric history and substance issues. Psychological status was assessed using the Chinese versions of the 24-item HAMD, Hamilton Anxiety Scale (HAMA), CES-D, and Ruminative Responses Scale (RRS). Depressive symptoms were measured with the CES-D and 24-item HAMD, anxiety symptoms with the HAMA, and ruminative symptoms with the RRS.

The Ethics Committee of the First Affiliated Hospital of Jinan University, China, approved the study (Approval No. 028, 2019). Participants provided informed consent after receiving a comprehensive written and verbal explanation of the study.

### 2.3 MRI data acquisition

Scanning was performed on a GE Discovery MR750 3.0T scanner(GE Healthcare, Waukesha, WI, USA) equipped with an 8-channel phased-array head coil to obtain rs-fMRI and 3D-BRAVO sequences. Complete acquisition parameters are detailed in the S1 Text.

### 2.4 Preprocessing of functional images

For functional image preprocessing, we employed the Data Processing Assistant for Resting-State fMRI (DPABI_V3.0) [26] toolbox, operating within the Statistical Parametric Mapping (SPM12, Wellcome Trust Centre for Neuroimaging, London, UK) environment. The preprocessing pipeline included: ① conversion of DICOM files to NIfTI format; ② removal of the first 10 volumes to allow for signal stabilization; ③ slice timing correction; ④ head motion correction; ⑤ spatial normalization to the MNI152 template using DARTEL with a resampling resolution of 3 × 3 × 3 mm^3^; ⑥linear detrending; ⑦ nuisance regression including 24 head motion parameters, white matter signal, cerebrospinal fluid signal, and global signal; and ⑧ band-pass filtering (0.01-0.1 Hz). Refer to the S1 Text for comprehensive details on the functional image preprocessing procedures.

**Fig 1:**
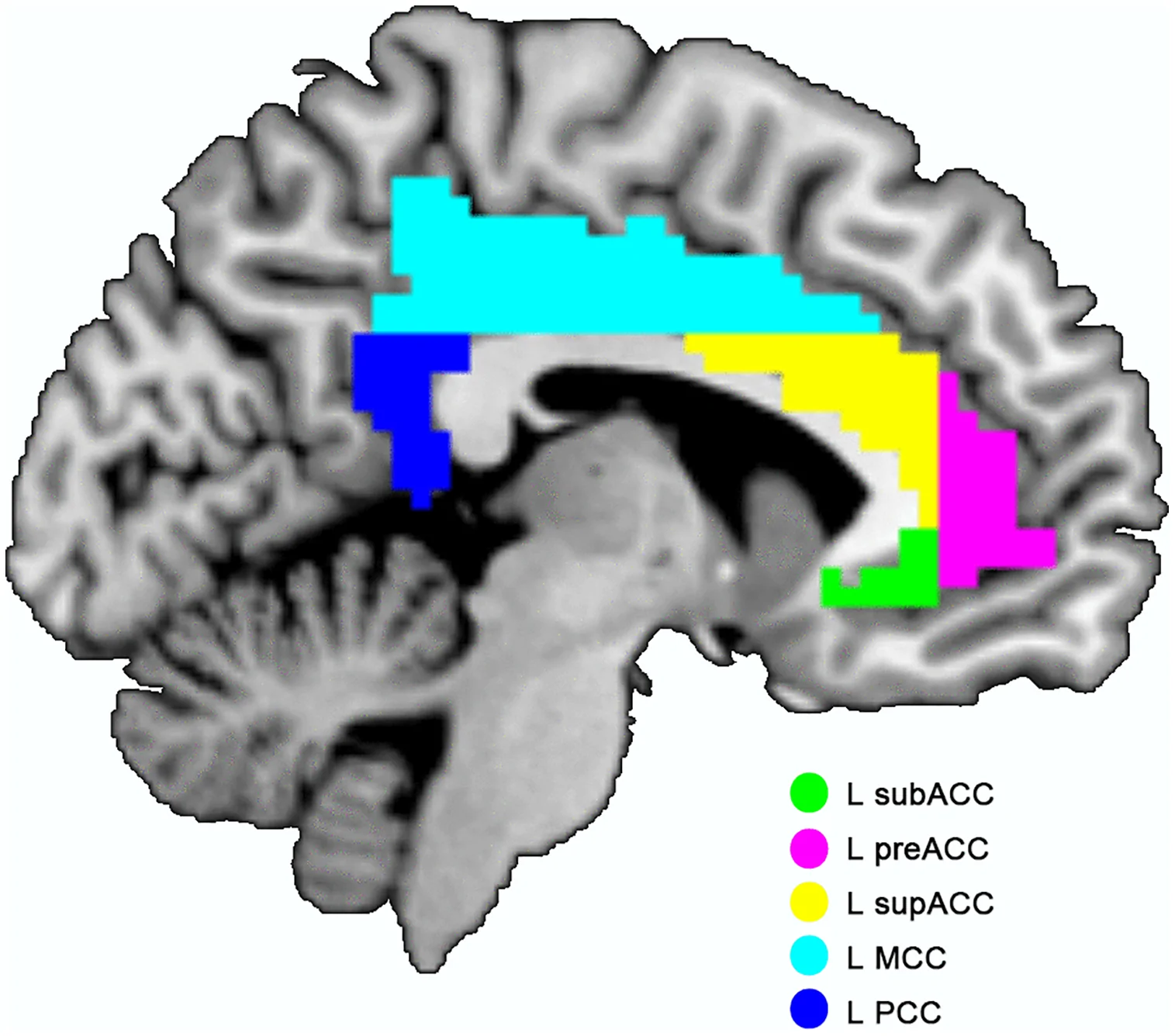
Five seeds of the cingulate cortex in the left hemisphere. subACC, subgenual anterior cingulate cortex; preACC, pregenual anterior cingulate cortex; supACC, supracallosal anterior cingulate cortex, MCC, middle cingulate cortex; PCC, posterior cingulate cortex; L, left.

Seed-based FC analyses were performed using regions of interest (ROIs) within the CC across ten masks. The cingulate cortex is functionally and anatomically heterogeneous and can be parcellated into distinct subregions with specialized roles in emotional, cognitive, and salience processing. Based on the Automated Anatomical Labeling (AAL) atlas and the methodology of Rolls et al. [27], we defined bilateral subgenual ACC (subACC), pregenual ACC (preACC), supracallosal ACC (supACC), middle cingulate cortex (MCC), and posterior cingulate cortex (PCC) as separate seeds. These subregions were selected because: [1] supACC is critically involved in negative emotion processing and cognitive control and is reliably altered in subthreshold depression; [2] MCC participates in affective regulation and pain-related affective processing; [3] PCC is a core node of the default mode network underlying self-referential and affective processing; [4] subACC and preACC are closely linked to reward, mood, and antidepressant treatment response. Collectively, these regions represent key hubs of emotion–cognition interactions and are highly relevant to the pathophysiology of depression. The anatomical locations of all ten cingulate subregional seeds are illustrated in Fig 1. Additional details on the FC analysis are provided in the S1 Text.

### 2.5 Cytokines measures

Fasting venous blood samples (4 mL) were drawn in the morning from both StD and HC subjects via venipuncture into sterile vacuum tubes. Participants were required to fast and avoid alcohol consumption for a minimum of 24 hours prior to sampling. Samples were clotted for 30 minutes, then centrifuged at 1,000 g for 15 minutes at 4°C. The serum underwent a second centrifugation at 3,000 rpm for 10 minutes at 4°C and was stored at -80°C until analysis. The serum was thawed on ice before testing, and the assay was conducted at room temperature following the kit's instructions to ensure optimal antigen-antibody binding. All samples were assayed in duplicate. Serum levels of pro-inflammatory cytokines TNF-α, IL-1β, IL-6 and IL-17 were measured using the Bio-Plex Pro Human Cytokine Assay kit and a Bio-Plex 200 array reader (Bio-Rad), according to the manufacturer's instructions. Data acquisition utilized Bio-Plex Manager Software version 6.1 ((Bio-Rad Laboratories, Hercules, CA, USA).

### 2.6 Statistical analysis

Demographic and clinical data between the two groups were compared using independent-sample t-tests in SPSS 24.0 (IBM Corp, Armonk, NY, USA). A *χ²* test was conducted to assess sex differences. The Mann-Whitney U test was employed to compare serum pro-inflammatory cytokine levels between groups, given the data's non-normal distribution. To stabilize variance and normalize the distribution, cytokine levels were natural log-transformed prior to analysis. Statistical tests were conducted as two-tailed with a significance level of *p* < 0.05. A one-sample t-test was performed on *z*-score maps for each mask to demonstrate the within-group FC spatial distribution of each seed for StD and HC within a brain mask. A two-sample *t*-*t*est assessed significant differences in whole-brain FC between StD and HC, using a combined mask from the one-sample *t*-*t*est results of each group. The whole-brain FC analysis included age, sex, education level, and mean framewise displacement for in-scanner head motion as nuisance covariates. Gaussian random field (GRF) theory was used for cluster-level multiple comparison correction, setting both voxel and cluster *p*-values to be below 0.005.

A Spearman correlation analysis was performed to evaluate the association between FC values and pro-inflammatory cytokine levels in the StD and HC groups, following the identification of significant differences in brain regions and cytokine levels. Spearman correlation coefficients were calculated to evaluate the associations between psychological variables, aberrant FC values, and elevated pro-inflammatory cytokine levels in the StD group. The psychological variables considered included CES-D scores, 24-item HAMD scores, HAMA scores, and RRS scores. The study utilized multiple linear regression to examine the relationships between FC values, psychological variables, and pro-inflammatory cytokine levels, while controlling for confounding variables such as sex, age, and education level. A *p*-value below 0.05 was established as the significance threshold.

## 3 Results

### 3.1 Demographic and psychological characteristics

Table 1 outlines the demographic and psychological profiles of the study participants. After excluding four StD and two HC participants for excessive head motion, the study included 126 StD and 104 HC participants. Statistical analysis indicated no significant differences in sex, age, education level, or FD parameters between the StD and HC groups (*p* > 0.05).

**Table 1:** Demographic and psychological data for StD and HC.

	StD	HC	*t/x*^*2*^ value	*p* value
Number of subjects	126	104	/	/
Sex (male/female)	48/78	43/61	0.252	0.616^c^
Age (years)	21.68 (2.84)	22.15 (3.30)	-0.50	0.618^a^
Education (years)	15.23 (2.38)	15.61 (2.68)	-1.139	0.256^a^
HAMD-24 score	14.10 (3.90)	2.09 (1.77)	28.333	<0.001^a^
CES-D score	29.86 (6.49)	9.56 (4.35)	27.106	<0.001^a^
HAMA score	14.71 (7.59)	1.43(1.88)	13.687	<0.001^a^
RRS score	52.72 (8.89)	32.74 (7.69)	17.407	<0.001^a^
FD	0.06 (0.03)	0.05 (0.03)	0.967	0.339^a^

HC, Healthy control; StD, Subthreshold depression; HAMD, Hamilton Depression Rating Scale; CES-D, Center for Epidemiologic Studies Depression Scale; RRS, Ruminative Responses Scale; HAMA, Hamilton Anxiety Scale; FD, framewise displacement of head motion.

Results are presented as means with standard deviations in parentheses, unless specified otherwise.

^a^The value was acquired by Independent-sample t-test.

^c^The sex distribution value was determined using a chi-square test.

### 3.2 Within-group FC distribution and between-group FC changes

**Fig 2:**
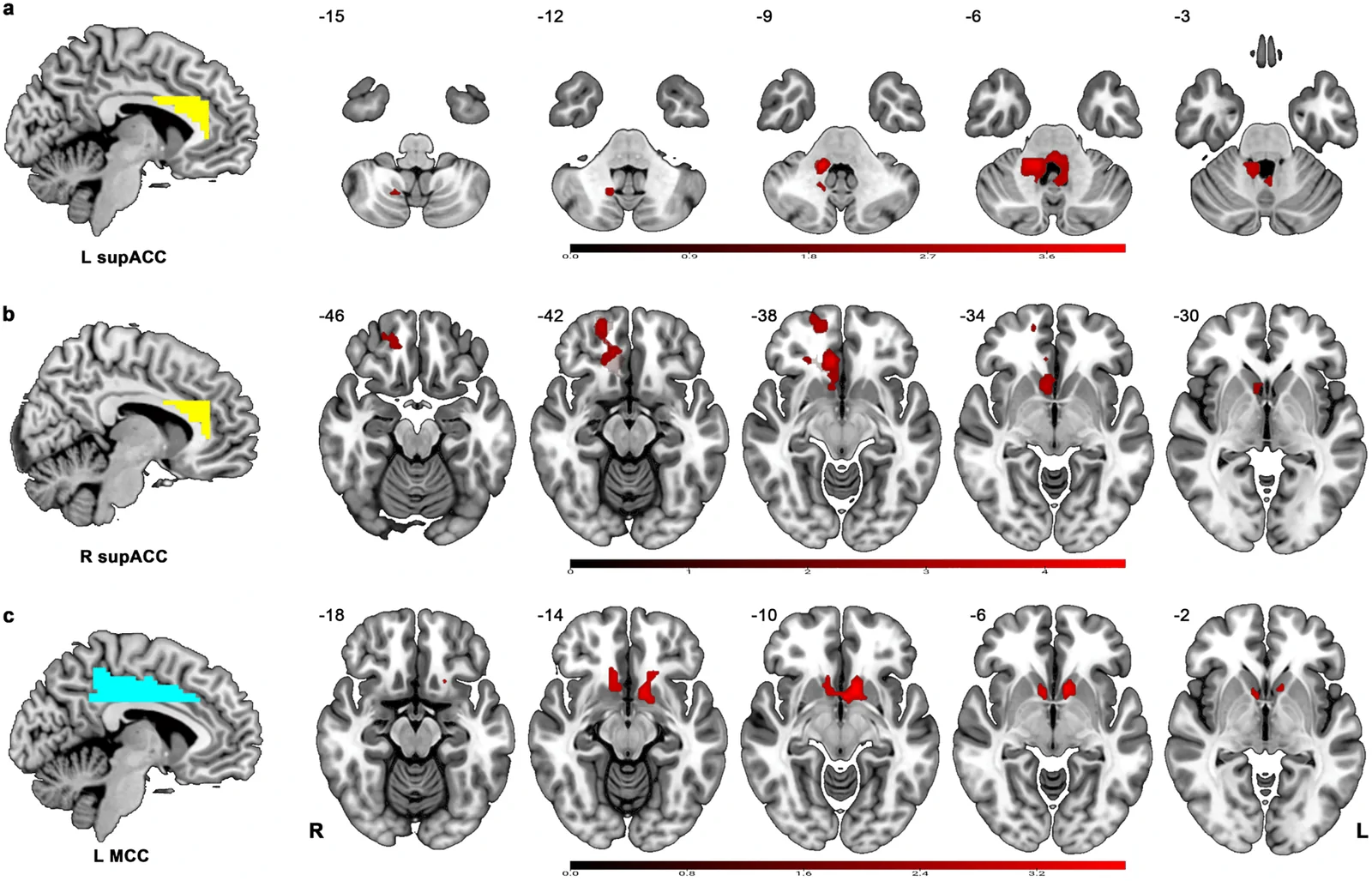
The whole brain FC differences in the cingulate cortex subdivisions between StD group and HC group. FC, functional connectivity; StD, Subthreshold depression; HC, healthy control; MCC, middle cingulate cortex; supACC, supracallosal anterior cingulate cortex, L, left; R, right.

A one-sample t-test assessed the within-group FC patterns in the StD and HC groups. Analysis indicated that the FC spatial distribution in the StD group was similar to that of the HC group (S1 Fig). The StD group showed enhanced FC compared to the HC group between the left MCC and caudate nucleus (CN), the left supACC and bilateral cerebellum anterior lobe, and the right supACC and right superior frontal gyrus (SFG). Conversely, no significant differences in FC were observed between the right/left subACC and other cerebral regions in the StD group relative to the HC group (refer to Fig 2 and Table 2).

**Table 2:** Regions showing notable FC differences between StD and HC groups (GRF correction, voxel p < 0.005, cluster p < 0.005).

Seeds	location in the cerebrum	Brodman Area (BA)	MNI Coordinates			Peak *t* Value	voxel numbers
X	Y	Z
L MCC	Bilateral Caudate	BA25	-3	6	-9	3.989	91
L supACC	Bilateral Cerebellum Anterior Lobe	/	12	-42	-36	4.256	135
R supACC	R Superior Frontal Gyrus	BA11	9	30	-12	4.737	157

StD, Subthreshold depression; HC, healthy control; FC, functional connectivity; GRF, Gaussian random field; MNI, Montreal Neurological Institute; MCC, middle cingulate cortex; supACC, supracallosal anterior cingulate cortex; L, left; R, right.

### 3.3 Group differences of pro‑inflammatory cytokines levels

Cytokine data was available for 75 participants with StD and 50 healthy controls. Table 3 compares TNF-α, IL-1β, IL-6 and IL-17 levels between the StD and HC groups. StD showed significantly elevated TNF-α, IL-1β and IL-17 levels compared to HC (*p* < 0.05), whereas IL-6 levels were not significantly different (*p* > 0.05). The missing data (51 StD and 54 HC) was due to the non-cooperation of the subjects and insufficient serum volume (n = 62), sample hemolysis (n = 28), and assay failure (n = 15).

**Table 3:** Pro‑inflammatory cytokines data for StD and HC.

	StD	HC	*t/x*^*2/*^*z* value	*p* value
Number of subjects	75	50	/	/
Sex (male/female)	21/54	16/34	0.230	0.691^c^
Age (years)	21.65 (2.71)	22.52 (3.06)	-1.624	0.108^a^
Education (years)	15.33 (2.41)	15.84(2.53)	-1.119	0.266^a^
IL-1β (pg/ml)	5.83 (5.20, 6.32)	5.53 (5.20, 5.87)	-2.157	0.031^b^
IL-6 (pg/ml)	12.27 (11.28, 13.08)	12.15 (11.09, 13.40)	-0.585	0.559^b^
IL-17 (pg/ml)	18.95 (17.65, 21.12)	18.22 (17.21, 19.23)	-2.394	0.017^b^
TNF-α (pg/ml)	130.25 (118.67, 144.24)	124.30 (114.31, 132.25)	-2.181	0.029^b^
Log IL-1β	1.76 (1.65, 1.84)	1.71 (1.65, 1.76)	-2.157	0.031^b^
Log IL-6	2.51 (2.42, 2.57)	2.50 (2.41, 2.59)	-0.585	0.559^b^
Log IL-17	2.94 (2.87, 3.04)	2.90 (2.85, 2.96)	-2.394	0.017^b^
Log TNF-α	4.86 (4.78, 4.97)	4.82 (4.74, 4.88)	-2.181	0.029^b^

StD, Subthreshold depression; HC, Healthy control; IL-1β, Interleukin-1β; IL-6, Interleukin-6; IL-17, Interleukin-17; TNF-α, Tumor necrosis factor-alpha; Log, natural logarithm.

Results are presented as means (standard deviations in parentheses) and medians (25% quartile, 75% quartile), unless specified otherwise.

^a^The value was derived using an independent-sample t-test.

^b^The value was derived using the Mann-Whitney U test.

^c^The sex distribution value was determined using a chi-square test.

### 3.4 Correlation analyses

**Fig 3:**
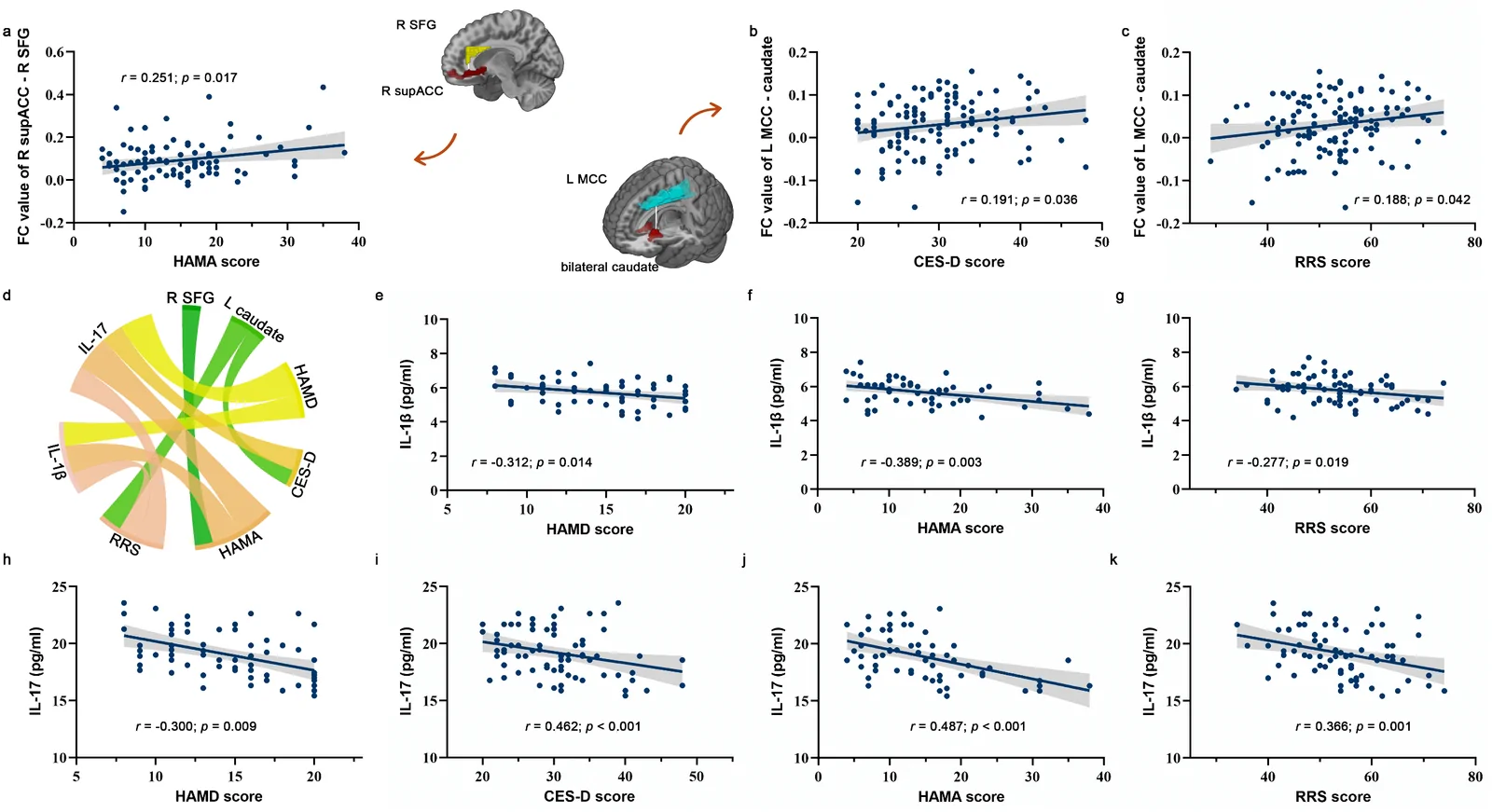
a-c, The correlation between altered FC and psychological variables in StD group. Fig 3d, Summary network of the correlation analysis. Fig 3e-k, The correlation between altered cytokines levels and psychological variables in StD group. Each scatter plot includes individual data points for all participants involved in the correlation analysis, with a fitted trend line indicating the correlation direction. HAMA, Hamilton Anxiety Scale; CES-D, Center for Epidemiologic Studies Depression Scale; RRS, Ruminative Responses Scale; HAMD, Hamilton Depression Rating Scale; FC, functional connectivity; supACC, supracallosal anterior cingulate cortex; SFG, superior frontal gyrus; MCC, middle cingulate cortex; IL-1β, Interleukin-β; IL-17, Interleukin-17; L, left; R, right.

The FC values in the left MCC - bilateral CN showed a positive correlation with CES-D scores (*r* = 0.191, *p* = 0.036), and RRS scores (*r* = 0.188, *p* = 0.042) in StD. The FC values in the right supACC - right SFG showed a positive correlation with HAMA scores (*r* = 0.251, *p* = 0.017) in StD (Fig 3a-c). In addition, the IL-1β level showed a negative correlation with the HAMD scores (*r* = -0.332, *p* = 0.008), HAMA scores (*r* = -0.389, *p* = 0.003), and RRS scores (*r* = -0.277, *p* = 0.019) in StD; and the IL-17 level showed a negative correlation with the CES-D scores (*r* = -0.300, *p* = 0.009), HAMD scores (*r* = -0.451, *p* < 0.001), HAMA scores (*r* = -0.487, *p* < 0.001), and RRS scores (*r* = -0.366, *p* = 0.001) in StD (Fig 3e-k). In StD, no significant correlations were found between FC values and cytokine levels. In the HC, there was no significant inter-relationships among psychological variables, cytokines levels and FC values (all *p* > 0.05). Fig 3d presents a summary of the correlation analyses.

### 3.5 Interaction of cytokines levels and FC values with psychological variables in StD and HC

Multiple regression analysis showed that the interaction between IL-1β levels and FC values in the right supACC - right SFG (*β* = -1.677, *t* = -2.543, *p* = 0.014) was an independent predictor of HAMA scores only in StD. Similarly, the interaction between IL-17 levels and FC values in the right supACC - right SFG (*β* = -3.214, *t* = -2.706, *p* = 0.009) independently predicted RRS scores exclusively in StD. In HC, cytokine levels interacting with FC values showed no significant correlation with psychological variables (*p* > 0.05).

## 4 Discussion

The study yielded the following findings: (1) The StD group demonstrated enhanced FC between CC and the bilateral caudate nucleus (CN), bilateral anterior cerebellar lobe, and right superior frontal gyrus (SFG). (2) Psychological characteristics in the StD group displayed significant alterations, reflected by elevated scores on CES-D, HAMD-24, HAMA, and RRS scales. In addition, the StD group exhibited increased levels of pro-inflammatory cytokines, including TNF-α, IL-1β, and IL-17. (3) In the StD group, FC values between the left middle cingulate MCC and bilateral CN displayed a positive correlation with CES-D and RRS scores. Furthermore, FC values between the right supACC and right SFG were positively correlated with HAMA scores. IL-1β levels depicted a negative correlation with HAMD, HAMA, and RRS scores, whereas IL-17 levels were negatively correlated with CES-D, HAMD, HAMA, and RRS scores. (4) The interaction between altered IL-1β levels and altered FC within the right supACC-right SFG circuit influenced HAMA scores in the StD group. Similarly, the interaction between altered IL-17 levels and altered FC within the right supACC-right SFG circuit influenced RRS scores. To the best of our knowledge, this is the first study to systematically examine associations among subregional cingulate functional connectivity, peripheral inflammatory markers, and clinical symptoms in individuals with subthreshold depression, with a specific focus on statistical interactions between peripheral cytokines and brain connectivity in relation to clinical symptoms. Although previous studies have explored either cingulate connectivity or inflammation in StD separately, the present study is the first to integrate these measures and demonstrate their combined associations with depressive and anxiety symptoms. These findings may improve understanding of the neural mechanisms underlying StD and depression from a neuroimmunological perspective.

### 4.1 Psychological characteristics and FC changes of StD

The study identified enhanced FC between the left MCC and bilateral CN in individuals with StD, and these FC values were positively correlated with CES-D and RRS scores. These findings suggest that enhanced connectivity between the bilateral CN and left MCC is associated with greater depressive and rumination symptoms. As a component of the basal ganglia, CN plays a central role in regulating emotion, cognition, and motor control [28]. Notably, previous research employing striatal seed-based FC analysis in the depressive population reported increased connectivity between CN and prefrontal cortex (PFC) [22]. This hyper-connectivity was significantly associated with depression severity. Furthermore, a trend toward an association between CN-PFC FC and RRS scores was observed. Another study demonstrated that, in late-life depression, regions exhibiting the strongest connectivity clusters with the right CN included the bilateral CC [29]. Butters et al. reported that reductions in CN volume were associated with depressive symptoms [30]. CN receives input from dopaminergic neurons originating in the ventral tegmental area. Multiple studies have demonstrated that reduced dopaminergic neurotransmission contributes significantly to depression pathophysiology [31]. Consequently, considering previous evidence supporting the association between impaired dopaminergic neurotransmission and CN atrophy in depression [32], dysfunction of the dopaminergic pathway may partially explain the relationship among depressive symptoms, CN dysfunction, and structural alterations. CN-MCC connectivity may be linked to emotional and cognitive processing in StD, consistent with prior observations in depression. The present findings further indicate that CN-MCC FC is associated with rumination, a maladaptive cognitive process characterized by repetitive negative thinking and persistent focus on distressing experiences. This FC pattern may reflect heightened emotional processing and may be associated with persistently low mood, a core characteristic of depressive symptomatology. Furman et al. [23] reported a trend for CN-PFC FC and ruminative thinking in depression, supporting substantial overlap between our findings in StD and established observations in major depression. Persistent involvement of limbic-caudate circuits in depression and StD may reflect their critical role in regulating cognitive processes related to reward processing, cognitive reappraisal, mood regulation, and reasoning, which contribute substantially to the clinical manifestations of these conditions. [33,34] These disruptions may represent neural correlates of early depressive symptoms. This finding could be utilized in the future to evaluate early stages of the disorder or individuals at risk [35].

Our analysis revealed that enhanced FC between the right SFG and the right supACC in individuals with StD exhibited a positive correlation with HAMA scores. The SFG is linked to several cognitive and emotional roles, such as planning complex behaviors, expressing personality, making decisions, moderating social interactions, and processing emotions [36,37]. In our prior investigation 16 utilizing functional MRI during a passive viewing task, we observed an enhancement in FC between the SFG and the CN during the processing of negative emotions in individuals with StD, as compared to HC. Our research group found that bright light therapy FC between the left ventral tegmental area (VTA) and the right SFG, with changes in VTA–SFG connectivity correlating with alterations in HAMD scores [38]. Huang et al. identified reduced spontaneous activity in the SFG in individuals with stress-related StD, highlighting the crucial role of SFG in the regulation of emotional processes within this context [39]. Reduced connectivity strength between the SFG and MCC was significantly associated with perceived stress levels in StD, highlighting the role of these regions in stress-related emotional dysregulation [37]. SFG activity has shown a positive correlation with anxiety in women and its connectivity with the PFC during tasks involving negative emotions [40]. Consequently, the existing literature indicates a complex pattern of SFG activity related to depression during the processing of negative emotions, which may vary depending on the nature of the behavioral tasks and the specific anxiety content. Notably, the activity of SFG appears to fluctuate across different emotion processing tasks, with emotion regulation tasks eliciting a greater SFG response compared to tasks involving passive exposure. Our results showing increased FC between the ACC-SFG, the correlation between FC and HAMA scores suggests that anxiety and depression have a statistical interaction on the functional changes of SFG.

The cerebellum is crucial for regulating muscle tension, maintaining body balance, and plays a significant role in emotion, language, working memory, and executive function [41]. Studies have identified structural and functional abnormalities in the cerebellum [42] of depression patients, encompassing gray matter volume [43], cerebral blood flow [44], glucose metabolism [45], functional activity [46], and connectivity [47]. Todeva-Radneva et al. identified enhanced FC between the left supACC and the bilateral cerebellum anterior on depression [48], which is consistent with ours finding on StD. A related study shows a positive correlation between the FC of the ACC and the cerebellum with HAMD scores in depressed individuals [49]. This suggests that cerebellar dysfunction may impede the integration of information required for mood state awareness, thereby hindering patients with cerebellar damage from explicitly recognizing their mood [50]. Our findings provide additional evidence for cerebellar dysfunction's involvement in early-stage depression pathophysiology.

### 4.2 Interaction of FC and pro-inflammatory cytokine changes in psychological characteristics in StD

This study found increased levels of pro-inflammatory cytokines TNF-α, IL-1β and IL-17 in StD, consistent with prior depression research [51]. Although we didn’t find significant correlations between cytokines and abnormal FC in brain regions, the interaction of altered IL-1β levels and altered FC of the right supACC - right SFG combine to influence the HAMA scores in StD. Cytokines are essential mediators in the interaction between the immune system and the brain, playing a key role in initiating and sustaining immune responses [52,53]. IL-1β acts as a critical driver in the pathogenesis of both neuroinflammation and depression [51]. Prior literature consistently reports elevated peripheral IL-1β concentrations in depressed patients [54–56]. Our results highlight a specific link between high IL-1β levels and anxiety symptoms, mediated by altered functional connectivity within the ACC-SFG circuit. Pandey et al.found significantly higher IL-1β levels in the prefrontal cortex of individuals with depression who died by suicide compared to control subjects [57]. A study showed that IL-1β levels of peripheral blood were the independent peripheral predictors of brain markers of inflammation in dorsolateral PFC expression in clinical high risk for psychosis by positron emission tomography (PET) scans [58]. In allergic rhinitis mice, notable anxiety and depression-like behaviors were observed, linked to ACC activation and elevated IL-1β levels, which further exacerbated these behaviors [59]. While the amygdala and hippocampus have traditionally been associated with anxiety control, we propose that the FC between the ACC and SFG may play a crucial role in depression comorbid with anxiety, potentially manifesting in the early stages of the condition. For the first time, we have linked changes in IL-1β to changes in brain function in StD, which can be inferred that higher IL-1β was associated with altered ACC–SFG connectivity and anxiety symptoms in StD.

Furthermore, we observed that the interplay between aberrant IL-17 levels and right supACC - right SFG connectivity significantly impacted RRS scores in StD subjects. As the primary member of a newly characterized inflammatory cytokine family, IL-17 serves as a vital early mediator in inflammatory responses induced by T-cells. It enhances the inflammatory cascade by stimulating the release of pro-inflammatory cytokines [60]. Several studies have demonstrated that in elderly patients suffering from coronary heart disease concomitant with depression, there is a marked increase in serum IL-17 levels, which correlates with the severity of depressive symptoms [61,62]. Subsequent studies have shown a positive correlation between pre-treatment serum IL-17 levels in depressed patients and the occurrence of negative life events, while no such association was found with positive life events [63]. Additionally, experiments conducted by Kühn and Cooney et al. have shown that the propensity for rumination in healthy individuals is linked to both the resting state activation and the ACC [64,65]. This change may be related to the increase of IL-17 in peripheral blood.

In conjunction with the aberrant expression of IL-1β and IL-17 observed in this study, it is proposed that StD may exhibit a predisposition towards autoimmune dysfunction. We suspect that IL-1β and IL-17 may show statistical interaction in inflammatory response and immune regulation, which co-influence the FC of ACC-SFG, causing rumination and anxiety. IL-1β and IL-17 may jointly relate to altered connectivity and clinical symptoms in StD.

An interesting and seemingly paradoxical finding is that IL-1β and IL-17 levels were significantly higher in the StD group than in HC, but negatively correlated with symptom severity within the StD group. This pattern may reflect an adaptive immune response in early-stage depression, where moderate inflammation triggers compensatory neuroprotective mechanisms; it may also indicate a nonlinear (U-shaped) relationship between inflammation and symptoms.

## 5 Limitation

Our study faced several limitations. The absence of a depression group limits our ability to generalize the observed brain activation changes to clinical depression, highlighting the need for direct comparisons between StD and depression. Additionally, the cross-sectional and retrospective design hindered the observation of progressive changes and the establishment of causal relationships among variables. A longitudinal study would enable comparisons across the continuum from StD to depression. Second, the sample consisted exclusively of right-handed university students of Han ethnicity recruited from Chinas. This highly homogeneous sample limits the generalizability of our findings to older adults, other ethnic groups, left-handed individuals, or non-student populations. Future studies should include more diverse samples to confirm the generalizability of our results. Third, considering that our study is exploratory, the results of our correlation analysis should be interpreted cautiously, as the results did not apply multiple comparison corrections. Fourth, the unexpected negative correlation between cytokines and symptoms within the StD group may reflect sampling variation, nonlinearity, outliers, or residual confounding. Finally, although we controlled for multiple potential confounders, unmeasured factors such as assess physical exercise, caffeine intake, sleep architecture, and hormonal status may still influence our results.

## 6 Conclusion

To conclude, this study demonstrates that StD is characterized by heightened FC between the ACC and SFG, a disruption likely associated with immune dysregulation as indicated by raised IL-1β and IL-17 levels. These findings highlight the critical involvement of compromised neuroimmune associations within the ACC-SFG circuit in the early neural correlates of depression.

## Supporting Information

S1 Fig The functional connectivity patterns within StD and HC groups. Shades of red represent increased/decreased dFC regions. FC, functional connectivity; subACC, bilateral subgenual anterior cingulate cortex; preACC, pregenual anterior cingulate cortex; supACC, supracallosal anterior cingulate cortex; MCC, middle cingulate cortex; PCC, posterior cingulate cortex; StD, subthreshold depression; HC, healthy control; R, right hemisphere; L, left hemisphere.
(TIF)

S1 Table Main effects of cytokine subgroup (participants with vs. without cytokine data) on clinical scales. HAMD, Hamilton Depression Rating Scale; CES-D, Center for Epidemiologic Studies Depression Scale; HAMA, Hamilton Anxiety Scale; RRS, Ruminative Responses Scale.
(DOCX)

S1 Data Clinical, demographic, and cytokine dataset. De-identified participant-level data including group assignment, age, sex, years of education, and cytokine measurements (IL-1beta, IL-6, IL-17, TNF-alpha). Key functional connectivity values extracted from significant clusters are also included. Missing values are indicated as blank cells. n = 230 (126 StD, 104 HC).
(XLSX)

S1 Text MRI data acquisition and functional image preprocessing protocols. (DOCX)
