# Supplementary material for: Inflammation is associated with altered functional connectivity of the cingulate cortex in individuals with subthreshold depression
Source: PLOS Digit Health. 2026 Sep 17;5(9):e0001665. doi: 10.1371/journal.pdig.0001665 (PMC13585250; doi:10.1371/journal.pdig.0001665)
Supplement: Supplementary file 1 [file pdig.0001665.s002.docx]

**Table S1.  Main effects of cytokine subgroup (with vs. without cytokine data) on clinical scales.**

| **Parameter** | ***F*** | ***P* value** |
| --- | --- | --- |
| HAMD score | 0.003 | 0.956 |
| CES-D score | 1.082 | 0.299 |
| HAMA score | 1.604 | 0.207 |
| RRS score | 0.773 | 0.380 |

HAMD, Hamilton Depression Rating Scale; CES-D, Center for Epidemiologic Studies Depression Scale; HAMA, Hamilton Anxiety Scale; RRS, Ruminative Responses Scale.
