# Supplementary material for: Inflammation is associated with altered functional connectivity of the cingulate cortex in individuals with subthreshold depression
Source: PLOS Digit Health. 2026 Sep 17;5(9):e0001665. doi: 10.1371/journal.pdig.0001665 (PMC13585250; doi:10.1371/journal.pdig.0001665)
Supplement: Supplementary file 2 [file pdig.0001665.s004.docx]

***S1***

**MRI Data Acquisition and Functional Image Preprocessing Protocols**

**Methods and Materials**

***MRI data acquisition***

The rs-fMRI and three-dimensional brain volume imaging (3D-BRAVO) data were obtained on a GE Discovery MR750 3.0T system with an 8-channel phased-array head coil. Participants were scanned in a supine, head-first position. To decrease head motion during the scan, two pairs of cushions were symmetrically placed on both sides of the head. Participants were informed to close their eyes in relaxation without falling asleep. After the scan, each participant was confirmed not having fallen asleep.

The rs-fMRI data were acquired using a gradient-echo echo-planar imaging (GE-EPI) sequence with the following parameters: time repetition (TR)/time echo (TE) = 2000/25 ms; flip angle = 90°; voxel size = 3.75 × 3.75 × 3 mm^3^; field of view (FOV) = 240 × 240 mm^2^; matrix = 64 × 64; slice thickness/gap = 3.0/1.0 mm; 35 axial slices covering the whole brain; and 210 volumes acquired in 7 min. And the 3D-BRAVO sequence covering the whole brain with the following parameters: TR/TE = 8.2/3.2 ms; flip angle = 12°; bandwidth = 31.25 Hz; slice thickness/gap = 1.0/0 mm; matrix = 256 × 256; FOV = 240 × 240 mm^2^; NEX = 1; and acquisition time = 3 min 45 s. In addition, routine MRI scans were also acquired for excluding any anatomic abnormality. All participants were confirmed with no brain structural abnormalities by two experienced neuroradiologists (ZQ and ZL, with 10 and 8 years of experience in neuroimaging, respectively).

***Functional image preprocessing***

The images were preprocessed through Data Processing Assistant for Resting-State fMRI (DPABI_V3.0, <http://restfmri.net/forum/DPABI)> (1), which is based on Statistical Parametric Mapping (SPM12, <http://www.fil.ion.ucl.ac.uk/spm/>). For each subject, the first 10 images of the rs-fMRI dataset were discarded to allow for magnetic stabilization. The remaining 200 images were first slice-time corrected and realigned to the first image for correcting for inter-TR head motion. This realignment correction provided a record of the head motion within the rs-fMRI scan. All subjects should have no more than 2 mm maximum displacement in any plane, 2° of angular motion as well as 0.2 mm in mean frame-wise displacement (FD) (2). The individual T1 structural images were segmented (white matter, gray matter, and cerebrospinal fluid) using a segmentation toolbox. Then, the DARTEL toolbox was used to create a study-specific template for accurate normalization. Then, resting-state functional images were co-registered to the structural images and transformed into standard Montreal Neurological Institute (MNI) space, resliced to a voxel size of 3 × 3 × 3 mm^3^ resolution. After resliced, the normalized functional images were removed linear trend and passed through band-pass filter of 0.01–0.1 Hz. Several spurious covariates (signals of the brain global mean, white matter, and cerebrospinal fluid) and temporal derivatives (Friston-24 parameters of head motion) were then regressed out from the time course of each voxel (3,4).

Following previous work (5), seed-based dFC analyses were performed by setting regions of interest (ROIs) of cingulate cortex (CC) within ten masks (Fig 1): bilateral subgenual ACC (subACC), bilateral pregenual ACC (preACC), bilateral supracallosal ACC (supACC), bilateral middle CC (MCC), bilateral posterior CC (PCC). The seed point reference time series of each CC subregion was calculated by extracting and averaging the time series over all voxels within itself. Individual FC maps of the CC subregions were generated by calculating Pearson’s correlation coefficients between the mean time series of the ROIs and the time series of each voxel in the whole brain. Subject-level correlation maps were then converted to z-value maps using Fisher’s r-to-z transformation to improve the normality. For all the subjects, two z-score maps which represented the intrinsic FC of the CC seeds were finally obtained. Finally, all the FC maps were smoothed using a 6mm full width at half maximum Gaussian kernel.

**References**

1. Yan CG, Wang XD, Zuo XN, Zang YF (2016): DPABI: Data Processing & Analysis for (Resting-State) Brain Imaging. Neuroinformatics 14: 339-351.
2. Jenkinson M, Bannister PR, Brady JM, Smith SM (2002): Improved optimization for the robust and accurate linear registration and motion correction of brain images. Neuroimage 17: 825-841.
3. Friston KJ, Williams S, Howard R, Frackowiak RSJ, Turner R (1996): Movement-related effects in fMRI time-series. Magn Reson Med 35: 346-355.
4. Power JD, Mitra A, Laumann TO, Snyder AZ, Schlaggar BL, Petersen SE (2014): Methods to detect, characterize, and remove motion artifact in resting state fMRI. Neuroimage 84: 320-341.
5. Rolls ET, Huang CC, Lin CP, Feng J, Joliot M (2020): Automated anatomical labelling atlas 3. Neuroimage 206: 116189.

**Table S1.  Main effects of cytokine subgroup (with vs. without cytokine data) on clinical scales.**

| **Parameter** | ***F*** | ***P* value** |
| --- | --- | --- |
| HAMD score | 0.003 | 0.956 |
| CES-D score | 1.082 | 0.299 |
| HAMA score | 1.604 | 0.207 |
| RRS score | 0.773 | 0.380 |

HAMD, Hamilton Depression Rating Scale; CES-D, Center for Epidemiologic Studies Depression Scale; HAMA, Hamilton Anxiety Scale; RRS, Ruminative Responses Scale.

**Figure Legends**

**S1 Fig. The functional connectivity patterns within StD and HC groups.** Shades of red represent increased/decreased dFC regions. FC, functional connectivity; subACC, bilateral subgenual anterior cingulate cortex; preACC, pregenual anterior cingulate cortex; supACC, supracallosal anterior cingulate cortex; MCC, middle cingulate cortex; PCC, posterior cingulate cortex; StD, subthreshold depression; HC, healthy control; R, right hemisphere; L, left hemisphere.
